# Epigastric Heteropagus Twin

**Published:** 2011-11-27

**Authors:** Muhammad Qasim, Mahmood Shaukat

**Affiliations:** Department of Paediatric Surgery, Mayo Hospital Lahore, Pakistan

**Keywords:** Conjoined twin, Monochorionic monoamniotic, Epigastric heteropagus twin

## Abstract

Parasitic twining is a rare type of monozygotic monochorionic monoamniotic asymmetrical conjoined twin. We report a case of epigastric heteropagus twin. An ultrasound scan showed a defect of 1.5 cm in the epigastrium. CT showed soft tissue lobulated mass with fat and air components coming out of the epigastric defect. At operation rudimentary alimentary canal with no viscera, was found in the parasite. The parasite was easily separated from the host.

## INTRODUCTION

Conjoined twins have expected frequency of 1 in 50000 to 100000 live births. Potter and Craig used the term of heteropagus for asymmetrical conjoined twins. Parasitic twins account for 1-2% of all conjoined twins. The dependent undeveloped twin, the parasite, is attached to independent developed twin called autosite at different sites. Parasite attached to host’s epigastrium is rare and called epigastric heteropagus [1, 2]. We are reporting a case of epigastric heteropagus twin to share the surgical findings.

## CASE REPORT

A full term male baby was born with an undeveloped parasite attached to epigastric region. He was brought to our hospital at the age of one month. The child was healthy weighing about 5 kg. The parents and other siblings were healthy.

On examination there was a 6x4 cm mass attached to the abdomen having rudimentary upper limbs, lower limbs, head and external genitalia (Fig. 1). An ultrasound scan showed a defect of 1.5 cm in the epigastrium through which the gut was herniating. CT scan showed a soft tissue lobulated mass with fat and air components coming out of the epigastric defect. There were no calcifications.

**Figure:**
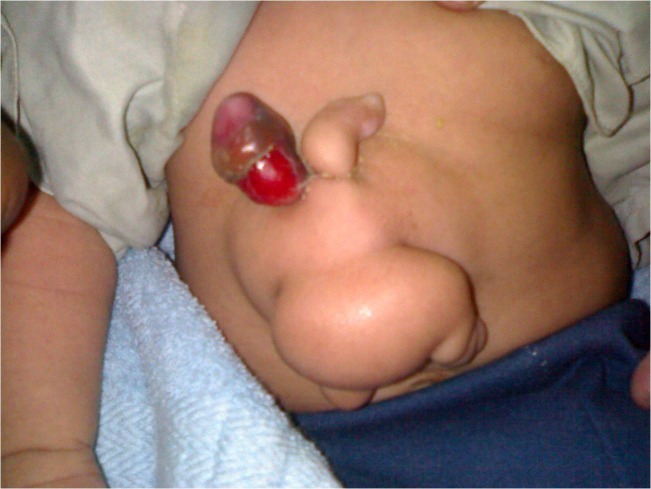
Figure 1: Parasite showing rudimentary limbs and phallus.

Separation of the parasite from the host was done easily. The parasite had soft tissue mass with no fully developed viscera. Rudimentary alimentary canal was present in the parasite (Fig. 2). There were no sharing of viscera between host and parasite. The postoperative recovery was uneventful and the baby was discharged on 8th postoperative day.

**Figure:**
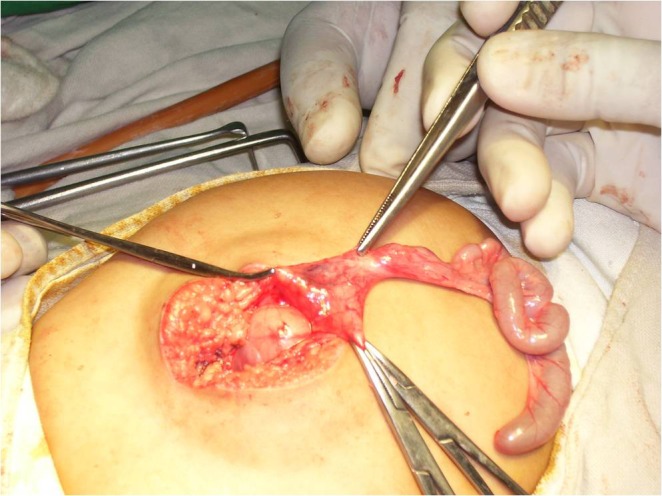
Figure 2: Rudimentary bowel of parasite.

## DISCUSSION

Conjoined twins are monozygotic twins in which the inner cell mass does not completely split. The two embryos are joined by a tissue bridge. Incomplete division of embryonic disk after 13th day post conception results in the formation of conjoined twins. Spencer proposed an alternative theory of fusion of two originally separate monozygotic embryonic disks, to explain the conjoined twin etiology. Some authors suggested that parasitic twin occur as a result of selective ischemic damage in-utero leading to death or partial resorption of, one of the twins, resulting in an incomplete parasitic twin attached to a fully developed twin [3, 4, 5].

Conjoined twin can be symmetrical or asymmetrical. Asymmetrical conjoined twins are called parasitic or heteropagus twins. It is further classified as

1- Externally attached parasitic twin

2- An enclosed fetus in fetu

3- An internal teratoma

4- Ancardiac connected via the placenta

The site and extent of twin fusion is extremely variable and the nomenclature is usually based on fused anatomical region as in this case the parasite was attached to the host in epigastric region so named as epigastric heteropagus [6, 7]. In our case, parasitic twin had rudimentary limbs and external genitalia. As in many of the reported cases, parasitic twin had limbs and trunk formed to variable extent but was acephalic and acardiac. In our case the blood supply of the parasite was from falciform ligament as noted in most reported cases [8, 9]. Epigastric heteropagus is a rare congenital malformation. The outcome and prognosis depends on the extent of visceral sharing and associated anomalies.

## Footnotes

**Source of Support:** Nil

**Conflict of Interest:** None declared
